# The Magnitude of *Candida albicans* Stress-Induced Genome Instability Results from an Interaction Between Ploidy and Antifungal Drugs

**DOI:** 10.1534/g3.119.400752

**Published:** 2019-12-11

**Authors:** Ognenka Avramovska, Meleah A. Hickman

**Affiliations:** Dept. of Biology, Emory University

**Keywords:** genome stability, fungal pathogen, ploidy, mutagenesis, antifungals

## Abstract

Organismal ploidy and environmental stress impact the rates and types of mutational events. The opportunistic fungal pathogen *Candida albicans*, serves as a clinically relevant model for studying the interaction between eukaryotic ploidy and drug-induced mutagenesis. In this study, we compared the rates and types of genome perturbations in diploid and tetraploid *C. albicans* following exposure to two different classes of antifungal drugs; azoles and echinocandins. We measured mutations at three different scales: point mutation, loss-of-heterozygosity (LOH), and total DNA content for cells exposed to fluconazole and caspofungin. We found that caspofungin induced higher mutation rates than fluconazole, although this is likely an indirect consequence of stress-associated cell wall perturbations, rather than an inherent genotoxicity. Surprisingly, we found that antifungal drugs disproportionately elevated genome and ploidy instability in tetraploid *C. albicans* compared to diploids. Taken together, our results suggest that the magnitude of stress-induced mutagenesis results from an interaction between ploidy and antifungal drugs. These findings have both clinical and evolutionary implications for how fungal pathogens generate mutations in response to antifungal drug stress and how these mutations may facilitate the emergence of drug resistance.

Mutations are the source material for adaptation. The mutational spectrum ranges from small-scale mutations, such as base substitutions and indels, to larger-scale mutational events such as gross chromosomal rearrangements and aneuploidy. Despite the presence of DNA repair mechanisms, all organisms incur mutations at a low level (Friedberg 2003); however, the rate of mutagenesis increases under stressful environments in both prokaryotes and eukaryotes (Tenaillon *et al.* 2004; Foster 2008; Petrosino *et al.* 2009; Forche *et al.* 2011; Maharjan and Ferenci 2017; Liu and Zhang 2019). For example, in yeast, the addition of inorganic salts, such as lithium chloride, increases the mutation rate by 3.5-fold (Liu and Zhang 2019). While stress increases mutational rates, it also shifts the mutational spectrum. Nutrient depletion, including phosphorus and nitrogen deficiencies, shifts the types of mutations from indels to base substitutions, as well as increasing the mutation rate by sevenfold in *E. coli* (Maharjan and Ferenci 2017). Taken together, stressful environments promote mutagenesis, thus generating more genetic variation for natural selection to ultimately act upon.

While environmental stress impacts the rates and types of mutation, it is not the only factor that influences the mutational landscape. Ploidy, the number of sets of chromosomes in an organism, is an important determinant of the mutational spectrum (Selmecki *et al.* 2015; Sharp *et al.* 2018). The opportunistic fungal pathogen, *Candida albicans* is a clinically-relevant model for studying the interaction between eukaryotic ploidy and stress-induced mutagenesis because of two key properties: the mutational rate and spectrum shift extensively when *C. albicans* is exposed to stress (Forche *et al.* 2011) and *C. albicans* exists across a range of ploidy states – from haploid to polyploid (Rustchenko 2007; Selmecki *et al.* 2010; Hickman *et al.* 2015; Todd *et al.* 2017). For example, *C. albicans* haploids potentially purge deleterious mutations that accumulate in diploids. However, there is a fitness cost associated with haploidy and/or the homozygous state, and haploidy is transient (Hickman *et al.* 2013). *C. albicans* is most-frequently isolated as a heterozygous diploid (Jones *et al.* 2004; Braun *et al.* 2005; Abbey *et al.* 2011; Muzzey *et al.* 2013) and can generate and maintain gross-chromosome rearrangements in laboratory and clinical isolates (Selmecki *et al.* 2009, 2010; Ford *et al.* 2015; Todd *et al.* 2017, 2019). Interestingly, large-scale genome mutational events, such as loss of heterozygosity (LOH), occur 1000-fold more frequently than point mutations (Forche *et al.* 2011). Tetraploid *C. albicans* can be isolated from patients (Suzuki *et al.* 1986; Legrand *et al.* 2004; Abbey *et al.* 2014), and generated in the laboratory through diploid-diploid mating or endoreplication (Forche *et al.* 2008). Tetraploids are pseudo-stable and undergo concerted chromosome loss, a process that generates cell heterogeneity through chromosome re-assortment, aneuploidy, and *SPO11*-dependent recombination (Hull *et al.* 2000; Magee and Magee 2000; Bennett and Johnson 2003; Forche *et al.* 2008; Hickman *et al.* 2015). Moreover, large-scale mutational events occur 30-fold more frequently in tetraploid *C. albicans* relative to the diploid state (Hickman *et al.* 2015). In summary, the mutational spectrums of haploid, diploid and tetraploid *C. albicans* are distinct, which is consistent with findings in other polyploid eukaryotes (Mayer and Aguilera 1990; Pavelka *et al.* 2010; Selmecki *et al.* 2015; Sharp *et al.* 2018).

As an opportunistic pathogen, *Candida albicans* is isolated in 40–60% of fungal infections in humans (Pfaller and Diekema 2007, 2010; Pfaller *et al.* 2008; Pfaller 2012; Perfect 2017) and encounters diverse stressors within its human host. *In vitro* stress-induced mutagenesis studies show that stressors such as febrile temperature (39°) elevate the rate of LOH and increase the frequency of whole-chromosome mis-segregation by ∼fivefold (Forche *et al.* 2011). *C. albicans* also encounters antifungal drugs, which are used to treat infection and include the azoles, which target the cell membrane, and echinocandins, which target the inner cell wall (Pappas *et al.* 2016; Perfect 2017). Antifungal drugs are potentially mutagenic, as fluconazole increases LOH rates (Forche *et al.* 2011) and generates aneuploidy (Harrison *et al.* 2014; Robbins *et al.* 2017; Stone *et al.* 2019), which is commonly associated with azole-resistance (Selmecki *et al.* 2006, 2009; Robbins *et al.* 2017; Stone *et al.* 2019). With only a limited number of drugs that effectively treat fungal infections (Pappas *et al.* 2016), a deeper understanding of drug-induced mutagenesis is necessary to understand the emergence of resistance.

While fluconazole-induced mutagenesis has been studied in diploid *C. albicans*, no study to date has examined how other types of antifungal drugs impact *C. albicans* genome stability, or how ploidy impacts this phenomena. In this study, we investigated the rates and types of mutations generated in diploid and tetraploid *C. albicans* exposed to two classes of antifungal drugs, echinocandins and azoles. We found that caspofungin elicited very high mutation rates regardless of ploidy, although this is likely an indirect consequence of stress-associated cell wall perturbations, rather than an inherent genotoxicity. Surprisingly, we found that antifungal drugs disproportionately elevated genome and ploidy instability in tetraploid compared to diploid *C. albicans*. These findings indicate that there is an interaction between ploidy and drug exposure that determines the magnitude of stress-induced mutagenesis. These results have evolutionary and clinically relevant implications for how fungal pathogens generate mutations that potentially drive the emergence of antifungal drug resistance.

## Materials and Methods

### Yeast strains and media

The strains used in this study are listed in Table S1. MH297 was constructed by replacing the *HIS4* open reading frame with the dominant drug-resistant *NAT* gene by lithium acetate transformation. Transformants were selected on YPD containing 50 µg/mL NAT and subsequently replica-plated onto media lacking histidine to verify candidates whose *HIS4* was knocked-out. MH296 resulted from mating two diploid *his4Δ*:: *NAT/his4-G929T*. All strains were stored as glycerol stocks at -80° and maintained on YPD (1% yeast extract, 2% bactopeptone, 2% glucose, 1.5% agar, 0.004% adenine, 0.008% uridine) at 30°. Antifungal drug treatments were made from the following stock solutions: 1 mg fluconazole (ACROS Organics CAS#86386-73-4) was diluted into 1 mL DMSO, 1 mg caspofungin (Sigma-Aldrich CAS#179463-17-3) was diluted into 1 mL ddH_2_0, and 10 mg calcofluor white (Sigma-Aldrich CAS#4404-43-7) was diluted into 1 mL ddH_2_0. Yeast cultures were grown in casitone (0.9% bacto-casitone, 0.5% yeast extract, 1% sodium citrate, 2% glucose) in the presence or absence of the drug treatments. Synthetic complete media (SDC; 0.17% yeast nitrogen base without amino acids or ammonium sulfate, 0.5% ammonium sulfate, 2 sodium hydroxide pellets, 0.004% uridine, 0.2% synthetic complete media, 2% agar, 2% glucose) was used for counting viable colony forming units (CFUs) following drug exposure.

### Drug susceptibility assay

The minimum inhibitory concentration (MIC) for diploid and tetraploid *C. albicans* was performed as previous described (Rosenberg *et al.* 2018) with slight modification. Briefly, single colonies were inoculated in YPD and incubated with shaking for ∼16hrs at 30°. Yeast cultures were subsequently normalized to 0.01 OD and 200μL was spread onto casitone plates containing 1% agar and left to dry for 10 min. Standardized E-test strips (Biomeureix) of fluconazole (0.016 µg/mL – 256 µg/mL) and caspofungin (0.002 µg/mL – 32 µg/mL) were placed upon the plates and incubated for 24 hr at 30° and then photographed.

### HIS4 reversion assay

The reversion rate of the *his4-G929T* allele was performed as previously described (Forche *et al.* 2011) with slight modification. Briefly, 10 single colonies of MH296 and MH297 were inoculated into 6 mL YPD and incubated with shaking for 24 hr at 30°. 1 mL of overnight cultures were aliquoted to 4 mL of the following media: no-drug, 1 µg/mL fluconazole, 10 µg/mL fluconazole, 0.25 µg/mL caspofungin, 2.5 µg/mL caspofungin, and 100 µg/mL calcofluor white. All treatments were incubated with shaking for 5 days at 30°. To determine the number of viable colonies for each treatment, 500 µL was taken from each culture for serial dilution and 100 µL of the 10^−4^ or 10^−5^ dilutions were plated onto SDC. To determine the number of revertants, the remaining 4.5 mL the cultures were harvested by centrifugation, washed with H_2_O, resuspended with 300 µL ddH_2_0 and plated onto large, 150mm × 15mm plates containing media lacking histidine. Plates were incubated for 48 hr at 30° before counting. The rate of reversion was determined by fluctuation analysis (Luria and Delbrück 1943). All experiments were performed in triplicate.

### GAL1 loss of heterozygosity assay

The rate of LOH at the *GAL1* locus was performed as previously described (Hickman *et al.* 2015). Briefly, 12 single colonies of MH84 (*gal1∆/GAL1*) and MH128 (*gal1∆/gal1∆/gal1∆/GAL1*) were inoculated into 2 mL casitone in the presence or absence of drugs and incubated with shaking for 24 hr at 30°. Cells were harvested by centrifugation, washed once with ddH_2_0, resuspended in 1 mL ddH_2_0 and serially diluted. To determine total cell viability for each treatment, 100 µL of the appropriate dilution was plated onto SDC (10^−5^ for no-drug; 10^−4^ for fluconazole; 10^−2^ for caspofungin and calcofluor white) and counted after 48 hr incubation at 30°. To select for cells that spontaneously lost *GAL1* during the 24 hr incubation, 100 µL of the appropriate dilution (10^−1^ for no-drug; 10^−1^ for fluconazole; 10° for caspofungin and calcofluor white) was plated onto 2-deoxygalactose (2-DOG; 0.17% yeast nitrogen base without amino acids, 0.5% ammonium sulfate, 0.004% uridine, 0.004% histidine, 0.006% leucine, 0.1% 2-deoxygalactose, 3% glycerol) and counted following 72 hr incubation at 30°. The LOH rates were determined by the method of the median (Lea and Coulson 1949) using the median estimator equation: $\frac{\tilde{r}}{m}-\ln\left(m\right)-1.24=0$.

All experiments were performed in triplicate. At least 48 single colonies from each treatment of MH84 and MH128 were picked from SDC and 2-DOG plates and stored as glycerol stocks at -80° for subsequent flow cytometry analysis.

### Flow cytometry assay

Single colony isolates obtained from SDC and 2-DOG following 24 hr drug treatment were analyzed for total DNA content using flow cytometry (Hickman *et al.* 2015).

Briefly, strains were inoculated into 900 µL YPD and incubated with shaking for ∼16 hr at 30°. 50 µL of the overnight cultures were re-inoculated into 450 µL fresh YPD and incubated for ∼6 hr. 200 µL of cells were harvested, washed with ddH_2_0, and resuspended in 20 µL 50:50 TE (50 mM Tris, pH 8; 50 mM EDTA). Cells were fixed with 95% ethanol and incubated overnight at 4°. The following day, cells were washed twice with 50:50 TE, resuspended with 50 µL RNAse A (1 mg/ml) and incubated for 1 hr at 37°. Cells were then collected via centrifugation and resuspended with 50µL proteinase K (5 mg/ml), and incubated for 30 min at 37°. Cells were subsequently washed with 50:50 TE, resuspended with 50 µL SybrGreen (1:100 dilution in 50:50 TE; Lonza, CAT#12001-798, 10,000X) and incubated overnight at room temperature, shielded from light. Cells were collected via centrifugation and resuspended with 150 µL 50:50 TE, briefly sonicated, and analyzed using an LSRII flow cytometer, with 10,000 events collected for each sample. To calibrate the LSRII, laboratory diploid (SC5314;(Gillum *et al.* 1984)) and tetraploid (mating product of RBY16 and CHY477; (Bennett and Johnson 2003)) strains were used and also served as internal controls. G1 peak values were determined by FITC-A intensity fit with the multi-Gaussian cell cycle model (FloJoV10). To assess the frequency of deviations in total DNA content, G1 peaks of experimental samples were compared to the mean G1 peak for either MH84 (n = 45) or MH128 (n = 48). Deviations to total DNA content were defined as any experimental sample with a G1 peak greater than 2 standard deviations from the mean G1 of its respective parental strain (MH84 or MH128).

### Statistical analysis

Statistical analysis was performed using GraphPad Prism 8 software. Data sets were tested for normality using the D’Agostino & Pearson omnibus normality test. To test for difference between the no-drug treatment and the drug treatments, we used the non-parametric, unpaired, Mann-Whitney *U*-test.

### Data availability

All strains are available upon request. The authors ensure that the required data for reproducing these findings and confirming the conclusions are available in the article and corresponding figures and tables. Supplemental material available at figshare: https://doi.org/10.25387/g3.8872604.

## Results

### C. albicans cell viability depends on ploidy and exposure to antifungal drugs

**Figure 1:**
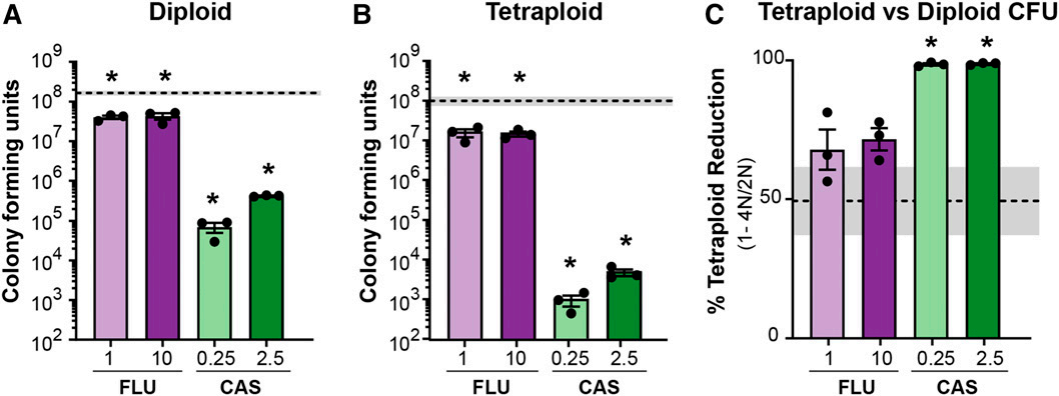
Diploid and Tetraploid *C.albicans* are sensitive to antifungals. A) Diploid colony forming units (CFUs) following 24-hr exposure to 1μg/mL or 10μg/mL fluconazole (‘FLU’, light and dark purple bars) and 0.25μg/mL or 2.5μg/mL caspofungin (‘CAS’, light and dark green bars) treatments. Bars represent the mean of 3 independent experiments (black symbols), and the error bars indicate +/− SEM. The dashed line and shaded box represent the mean and +/− SEM of the no-drug treatment. Asterisks indicate drug treatments that differ significantly from the no-drug treatment (* *P* < 0.05; ** *P* < 0.01; unpaired Mann-Whitney *U*-test). B) Tetraploid colony forming units. Data are displayed and analyzed the same as (A). C) The relative reduction of tetraploid CFUs compared to diploid CFUs. Data are displayed and analyzed similarly to (A).

To asses if antifungal drug susceptibility differs between diploid and tetraploid *C. albicans*, we determined the minimum inhibitory concentration (MIC) of fluconazole (FLU) and caspofungin (CAS) for an isogenic diploid and tetraploid pair. We observed no differences in drug susceptibility between the two ploidies and determined the MIC for fluconazole to be ∼1 µg/mL (Figure S1A) and for caspofungin to be less than 0.25 µg/mL (Figure S1B). To determine the cell viability after antifungal drug exposure, we measured diploid and tetraploid colony forming units (CFUs) following 24-hour exposure to antifungal drug treatments representing 1x and 10x the MIC (Figure 1A & B). While we detected minor, but statistically significant, CFU reductions following exposure to fluconazole compared to the no-drug treatment, we observed that growth rates in fluconazole were substantially slower (S1C & S1D). We found that exposure to caspofungin reduced CFUs more severely than fluconazole, in both diploid and tetraploid *C. albicans* (Figure 1A & B). Interestingly, the low concentration of caspofungin inhibited cell viablility to a greater degree than the high concentration, a phenomenon coined the “the paradoxical effect of echinocandins” (Wagener and Loiko 2017). Importantly, there were differences in total CFUs for diploid and tetraploid *C. albicans* in the no-drug treatment, where there were 50% fewer CFUs for tetraploids compared to diploids. We saw a similar reduction for fluconazole, where there were ∼50% fewer tetraploid CFUs than diploid. In contrast, caspofungin impacted tetraploid CFUs more severely, with a 90% reduction compared to diploid (Figure 1C). This demonstrates that antifungal drugs impact diploid and tetraploid *C. albicans* differently, despite having similar MICs and suggests there is a drug x ploidy interaction impacting cell viability.

### Drug x ploidy interactions impact the rates of genome instability

Given that short-term antifungal drug exposure impacted cell viability of diploid and tetraploid *C. albicans*, we next investigated if antifungal drug exposure increased mutation rates. Since mutations range from small-scale, (*i.e.*, single nucleotide mutations) to large-scale (*i.e.*, chromosome events including recombination and aneuploidy), we measured both point mutation and LOH rates in diploid and tetraploid *C. albicans* following exposure to fluconazole and caspofungin. To assess the point mutation rate, we used a *his4-G929T* (located on Chr4) reversion assay (Forche *et al.* 2011) and found that the reversion rate in the no-drug treatment was extremely low for both diploid (∼4 × 10^−10^ events/cell division) and tetraploid (∼12 × 10^−10^ events/cell division) *C. albicans*. Exposure to antifungal drugs had minor impacts on point mutation rates (Table S2). For example, exposure to high concentrations of fluconazole and caspofungin increased the tetraploid mutation rate only by twofold. Due to the rarity of revertants, and the fungicidal nature of caspofungin, it was technically challenging to capture enough reversion events to determine statistical significance.

**Figure 2:**
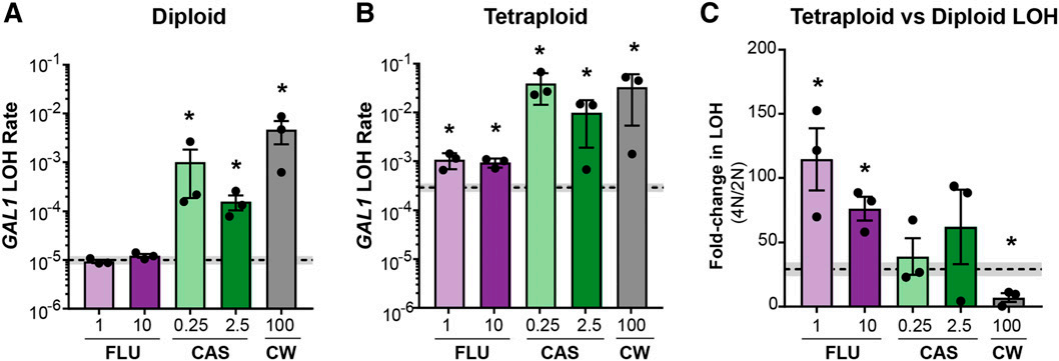
Ploidy and antifungal drug-specific impacts on LOH in *C. albicans*. A) Diploid *GAL1* LOH rates following 24-hr exposure to 1μg/mL or 10μg/mL fluconazole (‘FLU’, light and dark purple bars); 0.25μg/mL or 2.5μg/mL caspofungin (‘CAS’, light and dark green bars); and 100 μg/mL calcofluor white (’CW’, gray bar) treatments. Bars represent the mean of 3 independent experiments (black symbols), and the error bars indicate +/− SEM. The dashed line and shaded box represent the mean and +/− SEM of the no-drug treatment. Asterisks indicate drug treatments that differ significantly from the no-drug treatment (* *P* < 0.05; ** *P* < 0.01; unpaired Mann-Whitney *U*-test). B) Tetraploid *GAL1* LOH. Analysis was performed the same as in (A). C) The fold-increase in *GAL1* LOH rate in tetraploid relative to diploid *C. albicans*. Data are displayed and analyzed similarly to (A).

While point mutations are rare, large-scale genomic rearrangements occur frequently in *C. albicans* and are easily detected through loss-of-heterozygosity assays (Lea and Coulson 1949; Forche *et al.* 2011; Hickman *et al.* 2015). We found that diploid *C. albicans* exposed to caspofungin, but not fluconazole, showed significant increases in LOH rates compared to the no-drug treatment (Figure 2A). In contrast, for tetraploid *C. albicans*, exposure to either drug resulted in significant increases in LOH rates compared to the no-drug treatment (Figure 2B). However, the degree to which the specific antifungal drug elevated LOH rates differed: fluconazole increased the LOH rate by ∼threefold, whereas caspofungin increased the LOH rate by ∼100-fold.

We also observed significant differences in LOH rates between diploid and tetraploid *C. albicans* (Figure 2C). In the no-drug treatment, the rate of LOH was 1.0x10^−5^ events/cell division in the diploid state (Figure 2A, dashed line), whereas the tetraploid LOH rate was 2.9 x10^−4^ events/cell division (Figure 2B), a difference of ∼30-fold (Figure 2C), and consistent with previous reports (Hickman *et al.* 2015). If antifungal drugs elevate LOH rates to the same extent in both diploid and tetraploid *C. albicans*, then we hypothesized that there would also be a 30-fold difference across all drug treatments. Indeed, this is what we observed in caspofungin, where the tetraploid LOH rate was ∼40-fold higher than that of diploid (Figure 2C). In contrast, the fluconazole-induced LOH rate was 60-fold higher in tetraploids compared to diploids (Figure 2C). In addition to the individual contributions from ploidy (*P* = 0.0024, two-way ANOVA) and drug treatment (*P* = 0.0011, two-way ANOVA), we detected a significant interaction between these two variables (‘interaction’ *P* = 0.0019, two-way ANOVA; Figure S2) on LOH rates.

Regardless of ploidy, we observed that caspofungin significantly increased LOH rates, a surprising result since there is no evidence for its genotoxicity, yet some evidence that it induces apoptosis (Hao *et al.* 2013). To test whether the caspofungin-induced increase in LOH rates was due to genotoxicity of this drug, or simply an indirect consequence of cell wall stress, we exposed diploid and tetraploid *C. albicans* to calcofluor white, a cell-wall damaging agent (Walker *et al.* 2008). Importantly, both calcofluor white and caspofungin activate the *MKC1* pathway to elicit a downstream stress response (Munro *et al.* 2007; Walker *et al.* 2008; Heilmann *et al.* 2013). We found that at concentrations of 100 µg/mL, calcofluor white inhibited cell viability to a similar degree as 0.25 µg/mL caspofungin (Fig. S3) and elevated LOH rates in diploid (Figure 2A) and tetraploid (Figure 2B) *C. albican*s. The calcofluor white-induced LOH rates were comparable to what we observed in caspofungin, and suggests that caspofungin-induced LOH is an indirect consequence of cell wall stress rather than an inherent genotoxicity. Surprisingly, we saw that calcofluor white elicited a ploidy-specific impact on LOH rates, in which diploids were more severely impacted than tetraploids (Figure 2C). This was unexpected since caspofungin did not have any ploidy-specific responses.

### Antifungal exposure results in modest gains of total DNA content in diploids

**Figure 3:**
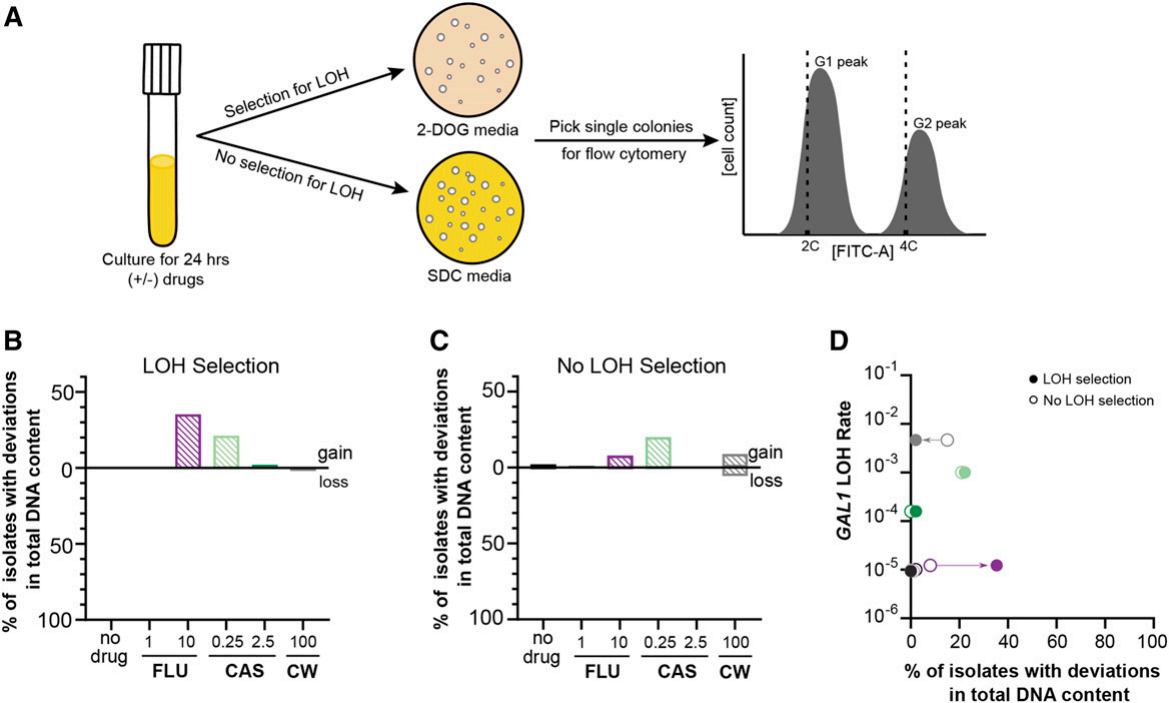
Diploids undergo small change in total genome size regardless of LOH selection. A) Experimental schematic. Cultures were incubated in the presence or absence of drugs for 24hrs and plated on 2-DOG to select LOH events or on SDC to determine determine total viable growth. At least 48 single colonies were picked from each plate type, prepared, and analyzed for flow cytometry. B) Frequency of diploid LOH derivatives (2-DOG) with deviations to total DNA content after 24-hr drug treatments. Isolates with G1 peaks greater than the 2C control were considered ‘gains’ in DNA content, and those with G1 peaks less than the 2C control were considered ‘losses’. C) Frequency of diploid isolates with deviations to total DNA in the absence of LOH selection (SDC) after 24-hr drug treatments. Data are represented similarly to (B). D) The frequency of deviations to total DNA content associated with the absence (open symbols) or presence (filled symbols) of selection for LOH events is plotted on the x-axis. The y-axis indicates the LOH rate for each drug treatment (represented by their respective colors).

Since exposure to antifungal drugs increased LOH rates, we examined whether there were corresponding changes in total DNA content. Following selection for LOH, we isolated single colonies and measured their total DNA content by flow cytometry (Figure 3A, 2-DOG). We found some changes to total DNA content following LOH selection (Figure S4). To quantify if these changes were gains or losses, we compared the G1 peak size of LOH derivatives to the mean G1 peak size of diploid controls. The no-drug, high caspofungin, and calcofluor white treatments did not elicit a meaningful frequency of LOH derivatives displaying changes to total DNA content (Figure 3B). However, high fluconazole and low caspofungin resulted in 35% and 20% of LOH derivatives displaying changes to total DNA content, respectively. Furthermore, when we detected changes, all were modest gains to DNA content (Figure 3B). These results suggest that chromosome mis-segregation is associated with fluconazole-induced LOH, consistent with previous findings (Forche *et.al*, 2011). Additionally, our results with caspofungin-induced LOH imply that most of these LOH events did not occur through chromosome mis-segregation mechanisms.

It is possible that the changes to total DNA content are a general response to antifungal drug stress, and not specifically associated with LOH events. Therefore, we measured total DNA content of single colony isolates obtained in the absence of LOH selection (Figures 3A, SDC and S4). The no-drug, low fluconazole, and high caspofungin treatments did not elicit a meaningful frequency of isolates displaying changes to total DNA content (Figure 3C). However, high fluconazole and low caspofungin resulted in 8% and 20% of isolates displaying gains in total DNA content, respectively. Calcofluor white resulted in ∼14% of isolates with changes to total DNA content, in which half were gains and half were losses (Figure 3C).

Next, we investigated whether there was a relationship between the frequency of changes to total DNA content (with and without LOH selection), and the mutagenicity of the specific drug treatment. To do this, we plotted the total fraction of isolates displaying changes to total DNA content (gains + losses) with LOH selection (Figure 3D, filled circles) and without LOH selection (Figure 3D, open circles) by the rate LOH for each drug treatment. If there is no relationship and these two variables are independent of each other, we hypothesize that there would not be a difference in the fraction of isolates with changes to total DNA content between selection regimes. Indeed, this is the case for the no-drug, caspofungin, and low fluconazole treatments (Figure 3D), despite the 100-fold increased LOH rate in caspofungin. While high fluconazole did not significantly alter the rate of LOH, there were high frequencies of isolates displaying total DNA content changes in the presence and absence of LOH selection. However, LOH derivatives had a substantially higher fraction of isolates displaying changes in total DNA content (Figure 3D, filled purple circle), suggesting that LOH events and changes to total DNA content were not independent in this treatment.

### Antifungal exposure results in drastic losses of total DNA content in tetraploids

**Figure 4:**
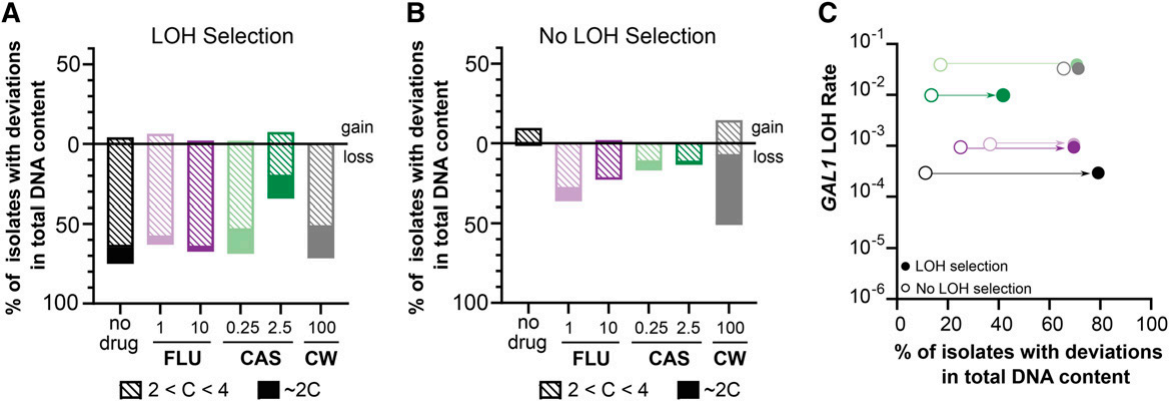
Tetraploids frequently exhibit substantial changes in total DNA content regardless of selection for LOH events. A) Frequency of tetraploid LOH derivatives (2-DOG) with deviations to total DNA content after 24-hr drug treatments. Isolates with G1 peaks greater than the 4C control were considered ‘gains’ in DNA content, and those with G1 peaks less than the 4C controls were considered ‘losses’. A small subset of isolates had G1 peaks that were within +/− 2SD of the 2C control and are indicated by the filled bars. B) Frequency of tetraploid isolates with deviations to total DNA in the absence of LOH selection (SDC) after 24-hr drug treatments. Data are represented similarly to (A). C) The frequency of deviations to total DNA content associated with the absence (open symbols) or presence (filled symbols) of selection for LOH events is plotted on the x-axis. The y-axis indicates the LOH rate for each drug treatment (represented by their respective colors).

While we observed a relationship between changes in total DNA content and LOH in high fluconazole for diploids, we wanted to determine whether antifungals affected this relationship in tetraploids. Following LOH selection, we isolated single colonies and measured total DNA content by flow cytometry. For tetraploids, we found substantial deviations in total DNA content occurring across all treatments (Fig. S4). We quantified these deviations similarly to our diploid analysis, and compared the G1 peak size of the LOH derivatives to tetraploid and diploid controls to categorize changes to total DNA content into two groups: 1) LOH derivatives with G1 peaks between 2C and 4C, or above 4C (indicating aneuploidy), and 2) LOH derivatives with ∼2C content, (indicating a halving of total tetraploid DNA content). LOH selection led to reductions in total DNA content, with 52–65% of LOH derivatives showing aneuploid genome contents for all treatments, except for high caspofungin, where only 20% are in this category (Figure 4A). Furthermore, a small number of LOH derivatives had DNA content at ∼2C, though this varied depending on drug treatment. For example, 2% of LOH derivatives were ∼2C in the high fluconazole compared to ∼18% in calcofluor white. These results support the model that tetraploids selected for LOH events are accompanied by reductions in DNA content (Hickman *et al.*, 2015).

While it is likely that the changes to total tetraploid DNA content are specifically associated with LOH events, these changes may be a general response to antifungal drugs. Therefore, we measured total DNA content of single colony isolates obtained in the absence of LOH selection (Figure S4). In the no-drug treatment, there were only a few minor deviations from tetraploidy, and a similar fraction detected in caspofungin, although these were mostly losses (Figure 4B). For fluconazole, 20–30% of isolates had aneuploid genome content and a small fraction had converged to diploidy. This supports the premise that fluconazole promotes ploidy instability, given that we also observed changes to DNA content in diploids. Interestingly, in calcofluor white, there were ∼40% of isolates with ∼2C DNA content. This rapid convergence to diploidy was notable given the short exposure time and absence of LOH selection. Furthermore, this was specific to calcofluor white and not a general response to cell wall perturbations, as we did not observe similar changes in caspofungin.

For each drug treatment, we plotted the total fraction of isolates displaying changes to total DNA content (gains + losses) with LOH selection (Figure 4C, filled circles) and without LOH selection (Figure 4C, open circles) by the rate of LOH. Deviations to total tetraploid DNA content were frequently (10–62%) detected in the absence of LOH selection across all treatments (Figure 4C), and is in contrast to diploids (Figure 3D). Furthermore, tetraploid instability was substantially elevated in isolates selected for LOH events. For no-drug and low caspofungin treatments, we saw a strong relationship between devations to total DNA content and selection for LOH; for fluconazole and high caspofungin treatments there was a moderate relationship; and for calcofluor white there was no relationship. This was due, in part, to the high ploidy instability observed in the absence of LOH selection and may also reflect LOH events that are not due to chromosome mis-segregation.

## Discussion

Both stress-induced mutagenesis and organismal ploidy impact mutation rates and spectra, but these two phenomena are often studied in isolation. Here, we investigated how antifungal drug stress impacts mutation rates of small- and large-scale genomic events in diploid and tetraploid *C. albicans*. We found a significant interaction between ploidy and antifungal drug stress on LOH rates (‘interaction’ *P* = 0.0160, two-way ANOVA, Figure S2). This was surprising, since we expected that the relative increase in drug-induced LOH rate would be similar between diploid and tetraploid *C. albicans*, despite inherent differences in their baseline rates. In fact, we observed this for caspofungin which substantially elevated genome instability for both ploidy states. However, fluconazole treatments impacted tetraploid *C. albcians* more severely than diploid *C. albicans*. Our findings support a model in which the magnitude of stress-induced mutagenesis is the result of a drug x ploidy interaction.

While *C. albicans* genomic responses to fluconazole has been well-studied (Forche *et al.* 2011; Harrison *et al.* 2014; Hickman *et al.* 2015; Popp *et al.* 2017), the genomic responses to caspofungin are largely uninvestigated, as most studies regarding caspofungin focus on mechanisms of resistance (Munro *et al.* 2007; Pfaller *et al.* 2008; Walker *et al.* 2008; Lee *et al.* 2012; Wiederhold 2016; Revie *et al.* 2018). Here, we found that caspofungin increased the rate of LOH (Figure 2) and point mutations (Table S2) for both diploid and tetraploid *C. albicans* compared to the no-drug and fluconazole treatments. Furthermore, exposure to caspofungin increased the frequency at which diploids and tetraploids changed total DNA content (Figure 3, 4 and S4). While an increase in caspofungin-induced genome instability was expected, given that it induces apoptosis (Hao *et al.* 2013), the degree to which this occurred was unexpected. These findings prompted us to test which is whether caspofungin is genotoxic or if the mutagenicity is an indirect result from stress to the cell wall. To test this, we exposed *C. albicans* to calcofluor white, a drug that interferes with the proper construction of the fungal cell wall (Walker *et al.* 2008) and found that genome instability was induced to a similar degree as caspofungin (Figure 2, 3 and 4) (Walker *et al.* 2008). This result suggests that caspofungin-induced mutagenesis is not due to its direct genotoxicity, but likely is an indirect consequence of perturbations to the cell wall. Intriguingly, caspofungin has a paradoxical effect on fungal cells, a phenomenon in which supra-MIC concentrations are permissible for proliferation, despite being susceptible at the MIC (Wagener and Loiko 2017). *C. albicans* restructures its cell wall at supra-MICs of caspofungin to better mediate this stress (Walker *et al.* 2008; Wagener and Loiko 2017). In our study we also saw the paradoxical effect of caspofungin in both diploid and tetraploid *C. albicans* viability and LOH rates (Figures 1 and 2). Our findings suggest that the magnitude of caspofungin-induced mutagenesis is dependent on its concentration, in which low concentrations signal a stronger stress response and elicit more genomic perturbations.

Given that there is a drug x ploidy interaction for LOH rates (Figure S2), we expected to see a similar relationship for how frequently changes to total DNA content occurred. Indeed, this is the pattern that we observed. Caspofungin increased the frequency of changes to total DNA content in both diploid and tetraploid *C. albicans* (Figures 3 and 4), consistent with its impact on LOH rates (Figure 2C). However, the directionality of genome size changes depended on ploidy. In diploids these changes were exclusively gains in DNA content, but in tetraploids they were predominantly losses. Fluconazole also elicited gains in DNA content in diploids (Figure 3) and losses in tetraploids (Figure 4). However, the frequency of these changes was substantially greater in tetraploids, indicative of a ploidy-specific response, consistent with the fluconazole-induced increase in tetraploid LOH rates (Figure 2C).

There is a relationship between LOH events and changes to total DNA content for diploids exposed to fluconazole (Forche *et.al*, 2011*)*, in which LOH events are predominantly associated with chromosome mis-segregation, and our findings also support this. Interestingly, this was not the case for caspofungin, where changes to total DNA content were not necessarily associated with selection for LOH, since high ploidy instability was observed even in the absence of LOH selection. For tetraploids, the co-occurrence of LOH and deviations in total DNA content is well established (Hickman *et al.*, 2015; Gerstein *et al.*, 2017), and our findings also support this. A majority of tetraploid LOH derivatives showed reductions in DNA content, regardless of drug treatment and in fact, the absence of drug resulted in the highest frequency of isolates with changes to DNA content. Therefore, the relationship between LOH and changes to DNA content is strongest in the no-drug treatment and weaker in the drug treatments (Figure 4C), and suggests that chromosome mis-segregation is not the major mechanism by which LOH occurs in tetraploids exposed to drugs. Taken together, we show that tetraploid *C. albicans* genomes are more unstable than diploids, and this instability is exaggerated upon antifungal drug exposure.

Tetraploid genome instability has been shown, both theoretically and experimentally, to facilitate rapid adaptation in yeast (Berman and Hadany 2012; Selmecki *et al.*, 2015), and this premise has important implications for the emergence of antifungal drug resistance. Resistance to antifungal drugs can result from point mutations, though the target genes differ between fluconazole and caspofungin (Revie *et al.* 2018). Furthermore, homozygosis of azole-resistance alleles (*i.e.*, LOH) substantially increases fluconazole MICs compared to heterozygous genotypes (Flowers *et al.* 2012; Ford *et al.* 2015). Likewise, aneuploidy is commonly associated with azole resistance (Selmecki *et al.* 2006, 2009; Robbins *et al.* 2017; Revie *et al.* 2018; Stone *et al.* 2019) and recent studies suggest that aneuploidy is beneficial for growth in caspofungin (Yang *et al.* 2019). Our study explicitly demonstrates that antifungal-induced genome instability impacts tetraploids more profoundly than diploids, and results in extremely high LOH rates and frequency of genome size changes, that are likely to contain aneuploid chromosomes. Thus, tetraploids have the capacity to provide an abundance of genetic variation for natural selection to act upon.
